# Identification of Potential Serum Metabolic Biomarkers of Diabetic Kidney Disease: A Widely Targeted Metabolomics Study

**DOI:** 10.1155/2020/3049098

**Published:** 2020-03-02

**Authors:** Hang Zhang, Jing-jing Zuo, Si-si Dong, Yuan Lan, Chen-wei Wu, Guang-yun Mao, Chao Zheng

**Affiliations:** ^1^Diabetes Center and Department of Endocrinology, The Second Affiliated Hospital and Yuying Children's Hospital of Wenzhou Medical University, No. 109 West Xueyuan Road, Wenzhou, China; ^2^Center on Clinical Research, School of Ophthalmology & Optometry, Wenzhou Medical University, Wenzhou, China; ^3^Center on Evidence-Based Medicine & Clinical Epidemiological Research, School of Public Health, Wenzhou Medical University, Wenzhou, China; ^4^The Second Affiliated Hospital of Zhejiang University School of Medicine, Hangzhou, China

## Abstract

*Background and Objectives.* Diabetic kidney disease is a leading cause of chronic kidney disease and end-stage renal disease across the world. Early identification of DKD is vitally important for the effective prevention and control of it. However, the available indicators are doubtful in the early diagnosis of DKD. This study is aimed at determining novel sensitive and specific biomarkers to distinguish DKD from their counterparts effectively based on the widely targeted metabolomics approach. *Materials and Method.* This case-control study involved 44 T2DM patients. Among them, 24 participants with DKD were defined as the cases and another 20 without DKD were defined as the controls. The ultraperformance liquid chromatography-electrospray ionization-tandem mass spectrometry system was applied for the assessment of the serum metabolic profiles. Comprehensive analysis of metabolomics characteristics was conducted to detect the candidate metabolic biomarkers and assess their capability and feasibility.

**Result:**

A total of 11 differential metabolites, including Hexadecanoic Acid (C16:0), Linolelaidic Acid (C18:2N6T), Linoleic Acid (C18:2N6C), Trans-4-Hydroxy-L-Proline, 6-Aminocaproic Acid, L-Dihydroorotic Acid, 6-Methylmercaptopurine, Piperidine, Azoxystrobin Acid, Lysopc 20:4, and Cuminaldehyde, were determined as the potential biomarkers for the DKD early identification, based on the multivariable generalized linear regression model and receiver operating characteristic analysis.

**Conclusion:**

Serum metabolites might act as sensitive and specific biomarkers for DKD early detection. Further longitudinal studies are needed to confirm our findings.

## 1. Introduction

Type 2 diabetes mellitus (T2DM) affects over 366 million people worldwide (6.4% of the adult population) and this number is expected to rise to 552 million by 2030 [1]. As the disease progresses, diabetes can be complicated by a series of diseases, in which diabetic kidney disease (DKD) is one of the most common microvascular complications [2]. DKD is also a major cause of chronic kidney disease and end-stage renal disease (ESRD) across the world, accompanied by an increased risk of mortality and cardiovascular disease. With economic growth and lifestyle changes, there are more and more T2DM patients at risk of progressive renal function loss.

Renal disease in diabetic patients is characterized by functional and structural abnormalities. Within the glomeruli, there is thickening of basement membranes, mesangial expansion, hypertrophy, and glomerular epithelial cell (podocyte) loss. In conjunction, the disease progresses in the tubulointerstitial compartment, leading to the expansion of tubular basement membranes, tubular atrophy, interstitial fibrosis, and arteriosclerosis [3, 4]. A large number of studies confirmed that hyperglycemia is the most important risk factor for DKD. Hyperglycemia promotes mitochondrial electron transport chain to generate excessive reactive oxygen species (ROS) through the formation of advanced glycation end products (AGEs) and the activation of the polyol pathway, hexosamine pathway, protein kinase C (PKC), and angiotensin II. Furthermore, the ROS initiates or enhances the oxidative stress and eventually results in the inflammatory response and formation of fibrosis. In addition, lipid metabolism abnormality, renin-angiotensin-aldosterone system (RAAS) activation, systemic and glomerular hypertension, insulin signaling impairment, increased growth factors and proinflammatory cytokines, and intracellular signaling pathway activation are also involved in the occurrence and progression of DKD [5].

The characterization of DKD develops silently in the clinical stage. DKD is featured by the initial appearance of microalbuminuria (MA) with a progressive increase in proteinuria and a decline in estimated glomerular filtration rate (eGFR). MA is often the first clinical sign of kidney involvement to predict overt nephropathy [6]. However, MA is suspected to result from such external factors as exercise, urinary tract infections, acute illness, and heart failure. eGFR, an indicator calculated from serum creatinine concentration, is also limited by the changes in creatinine production depending on age, gender, race, and body composition. In addition to poor specificity, its sensitivity in the prediction of DKD has also been questioned. Relevant studies have revealed that DKD tissue lesions are possible to precede MA significantly [6]. About only 35-45% of T2DM patients with MA will develop DKD in the next 6-10 years, of whom around 1/3 patients will spontaneously return to the state with normal albuminuria [7]. The early identification and treatment of DKD are conducive to lowering the risk of kidney damage by as much as 50%. Thus, it is essential to improve the ability to detect asymptomatic renal dysfunction and find more sensitive and specific biomarkers of DKD for early diagnose and predict the risk of DKD progression.

Metabolomics, which refers to the systematic and comprehensive analysis of metabolites (i.e., sugars, amino acids, organic acids, nucleotides, bile acids, acylcarnitine, and lipids) in a biologic sample, has been identified as a powerful tool in the biomarker discovery field [8–10]. Currently, there are several approaches being applied in metabolomics, such as nuclear magnetic resonance (NMR) [11], gas chromatography-mass spectrometry (GC-MS) [12], liquid chromatography-mass spectrometry (LC-MS) [13], and capillary electrophoresis mass spectrometry (CE-MS) [14]. Metabolomics approaches are especially promising in terms of nephrology research because that the extensive impact on kidney function has manifested on circulating metabolite levels, and metabolites possibly play functional roles in DKD pathogenesis and its complications. Targeted profiling, which is the detection of only a few sets of metabolites, has been used to identify biomarkers for DKD. Xia et al. conducted an analysis of six intermediate metabolites of the purine degradation pathway in plasma among patients with and without DKD using LC-MS [15]. It was discovered that adenosine, inosine, uric acid, and xanthine were powerful biomarkers to keep track of DM progression. Jiang et al. used high-performance liquid chromatography–electrospray tandem mass spectrometric (HPLC–ESI-MS/MS) for simultaneous quantification of eight aminothiols in the homocysteine metabolic cycle in plasma and identified two sulfur-containing metabolites, namely S-adenosylmethionine and S-adenosylhomocysteine, as the potential biomarkers for DM and DKD [16]. However, there are still few comprehensive metabolome profiles published of DKD. Therefore, the widely targeted metabolomics approach using ion-pair reversed-phase ultraperformance liquid chromatography-tandem mass spectrometry (UPLC-MS/MS) was taken in our study to investigate the variations of serum metabolites among DM patients with and without DKD for its early recognition.

## 2. Material and Method

### 2.1. Study Subjects

In the current study, 44 T2DM patients were recruited from the Second Affiliated Hospital of Wenzhou Medical University (WMU) based on their urine protein levels. The protocol has been carefully reviewed and approved by institutional review boards at the hospital before the study (LCKY2019-08). The procedures strictly followed the tenets of the Declaration of Helsinki, and all participants gave informed written consent before they participated in this study.

### 2.2. Study Population

The study was set up as a case-control study in the Diabetes Center and Department of Endocrinology, the Second Affiliated Hospital and Yuying Children's Hospital of Wenzhou Medical University. The diagnosis of T2DM in our study complied with the criteria set out by the American Diabetes Association [17]. The diagnostic criteria for CKD were renal structural impairment and dysfunction caused by a variety of factors for at least 3 months, including normal and abnormal glomerular filtration rate (GFR), pathological damage, blood or urine components, abnormalities, and imaging abnormalities, or an unexplained GFR < 60 mL/min/1.73 m^2^ that persisted for 3 months. The patients with infections or kidney stones, thyroid disease, current use of steroids, nephrotoxic drugs, angiotensin-converting enzyme (ACE) inhibitors, or angiotensin receptor blockers (ARBs) were excluded. The inclusion criteria are as follows: for all patients diagnosed with T2DM complicated with CKD but not undergoing renal replacement therapy (e.g., hemodialysis, peritoneal dialysis, and kidney transplantation) [18]. Participants were recruited and then divided into two groups: T2DM group without nephropathy (non-DKD, UACR (urinary albumin/creatinine ratio) < 3 mg/mmol, *n* = 20) and DKD group with microalbuminuria and macroalbuminuria (DKD, UACR > 3 mg/mmol, *n* = 24).

### 2.3. Sample Collection and Storage

After a minimum of 8 hours of fasting under complete aseptic precautions from all the subjects in the morning, blood samples (6 mL) were collected into tubes. Once the collection was completed, centrifugation took place at 716 × g for 10 minutes within 3 hours to separate the serum. All of the serum samples were frozen at -80°C before sample preparation.

### 2.4. Sample Preparation and Extraction

All the samples were thawed on ice for extraction of metabolites. 50 *μ*L of serum was put into 150 *μ*L of ice-cold methanol. After being stirred up sufficiently, the solutions were centrifuged at 25759 × g for 10 minutes at 4°C. Subsequently, the supernatant was collected and centrifuged at 25759 × g at 4°C for 5 minutes. Finally, the supernatant was derived for UPLC-MS/MS analysis.

### 2.5. UPLC Conditions

Combining ultraperformance liquid chromatography (UPLC, Shim-pack UFLC SHIMADZU CBM30A system, https://www.shimadzu.com/) and tandem mass spectrometry MS (QTRAP® 6500+ System, https://sciex.com/), an analysis was performed of the sample extracts with the use of an LC-ESI-MS/MS system. The conditions to facilitate the analysis were as follows: UPLC column, Waters ACQUITY UPLC HSS T3 C18 (1.8 *μ*m, 2.1 mm∗100 mm); column temperature, 40°C; flow rate, 0.4 mL/minutes; injection volume, 2 *μ*L; solvent system, water (0.04% acetic acid): acetonitrile (0.04% acetic acid); gradient program, 95 : 5 *V*/*V* at 0 minutes, 5 : 95 *V*/*V* at 11.0 minutes, 5 : 95 *V*/*V* at 12.0 minutes, 95 : 5 *V*/*V* at 12.1 minutes, and 95 : 5 *V*/*V* at 14.0 minutes.

### 2.6. ESI-Q TRAP-MS/MS

By applying a triple quadrupole-linear ion trap mass spectrometer (Q TRAP), QTRAP® 6500+ LC-MS/MS System, which is fitted with an ESI Turbo Ion-Spray interface, operates in positive and negative ion mode and is controlled by Analyst 1.6.3 software (AB Sciex); LIT and triple quadrupole (QQQ) scans were obtained. The ESI source operation parameters included the following: source temperature 500°C; ion spray voltage (IS) 5500 V (positive), -4500 V (negative); ion source gas I (GSI), gas II (GSII), and curtain gas (CUR) were set at 55, 60, and 25.0 psi, respectively; the collision gas (CAD) was high. With 10 and 100 *μ*mol/L polypropylene glycol solutions in QQQ and LIT modes, instrument tuning and mass calibration were conducted. Based on the metabolites eluted within this period, a specific set of MRM transitions were kept track of for every single period.

### 2.7. Data Processing and Analysis

After the serum metabolites assessment, the UPLC-ESI-MS/MS data were acquired by Analyst® Software 1.6.3 (AB Sciex), preprocessed (conversion, peak detection, retention time correction, and peak alignment) by MultiQuant™ Software (AB Sciex), and processed using MetaboAnalyst 4.0 (https://www.metaboanalyst.ca/) 25 and STATA MP 15.0 (Stata Corp, College Station, Texas, USA). Normalization was carried out by using sum and Pareto scaling (mean-centered and divided by the square root of the standard deviation of each variable) to standardize the data and make the features more comparable.

Differential metabolites between the 24 cases and 20 controls were detected using principal component analysis (PCA), orthogonal partial least squares discriminant analysis (OPLS-DA), fold change (FC) analysis, and Student's *t*-test, respectively. Furthermore, both a 10-fold crossvalidation test and a 1000-times permutation test were also carried out. The additional false discovery rate (FDR) method was applied to make adjustment for the testing of multiple hypothesis and the mitigation of false positives. The criteria of differential metabolite determination are as follows: *q* value (FDR adjusted *p* value) < 0.05 and FC value (the ratio of case/control) > 1.2 or <0.8, as well as variable importance in the project (VIP) > 1. Subsequently, multivariable generalized linear regression models (GLMs) were applied to comprehensively investigate the associations between each differential metabolite and the odds of DKD. Besides, the receiver operating characteristic (ROC) analysis was conducted to assess the value of the early detection on DKD with the detected metabolites-based biomarkers. All statistical tests were two-sided, and *p* ≤ 0.05 was treated as a significant level.

## 3. Result

### 3.1. Characteristics of the Study Participants

An analysis was performed of serum metabolic profiles from 44 patients in two groups.  Table 1 shows the clinical characteristics. The DKD group exhibited a lower level of high-density lipoprotein cholesterol (HDL-C) as compared to the non-DKD group. The other clinical parameters did not differ significantly between the two groups.

### 3.2. Serum Metabolome Profiles and Analysis with DKD

**Figure 1:**
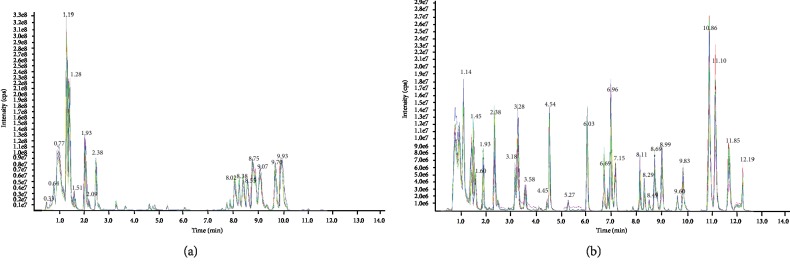
Typical TIC chromatograms obtained from the same serum sample of a DKD patient with (a) positive and (b) negative mode.

**Figure 2:**
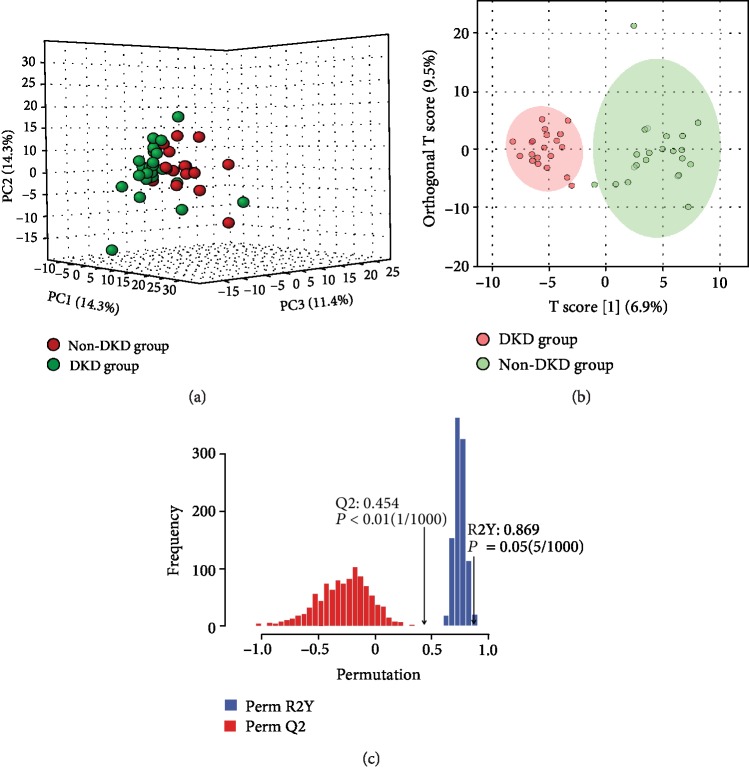
(a) Score plot of the PCA model. (b) Score plot of the OP-LSDA model (R2Xcum = 70%, R2Ycum = 83%, Qcum2 = 56%) showed the separation of the DKD group and non-DKD group. (c) 1000-times permutation test of the model showed that the model had high stability.

UPLC-ESI-MS/MS was detected from each serum sample. The complexity of the serum samples made the separation very difficult, thus resulting in severe ion suppression. UPLC employs the column with a smaller size of stationary phase particle size column, to generate high efficiency for the separation, which concurrently increased resolution and sensitivity. The *i*th redundant peaks were eliminated, such as noise, fragments, and adductions, and 613 serum metabolites remained. In this study, UPLC was applied to obtain the metabolic profiles in the positive and negative modes. A typical total ion chromatogram (TIC) of the serum of DKD patients for both ionization modes is shown in Figure 1. Based on the metabolites, we performed PCA, but the resultant score plots of the PCA showed no clear separation (Figure 2(a)). Then, OPLS-DA was used to demonstrate the satisfactory separation of DKD patients from non-DKD patients. One predictive component and one orthogonal component (R2Xcum = 70%, R2Ycum = 83%, Qcum2 = 56%) were involved in the OPLS-DA model, which showed that the model had a high stability (Figure 2(b)). In addition, the OPLS-DA model was further validated by means of permutation test (Figure 2(c)). The 24 cases and 20 controls were observed to be separated completely in the OPLS-DA score scatter plot, suggesting that the serum metabolic signatures can be effective in distinguishing between DKD patients from non-DKD patients.

### 3.3. Screening of Differential Metabolites

Of the metabolites in the OPLS-DA, according to the VIP values and *p* values (VIP > 1 and *p* < 0.01), a total of 11 candidate metabolites (Table 2) were discovered to be significantly different between DKD and non-DKD groups, suggesting the highly significant associations with DKD. They are Hexadecanoic Acid (C16:0), Linolelaidic Acid (C18:2N6T), Linoleic Acid (C18:2N6C), Trans-4-Hydroxy-L-Proline, 6-Aminocaproic Acid, L-Dihydroorotic Acid, 6-Methylmercaptopurine, Piperidine, Azoxystrobin Acid, Lysopc 20:4, and Cuminaldehyde. Among these metabolites, the most significant effect was observed for Linolelaidic Acid (C18:2N6T) (VIP = 6.25; *p* < 0.001).

### 3.4. Heatmap and Pathway Analysis

**Figure 3:**
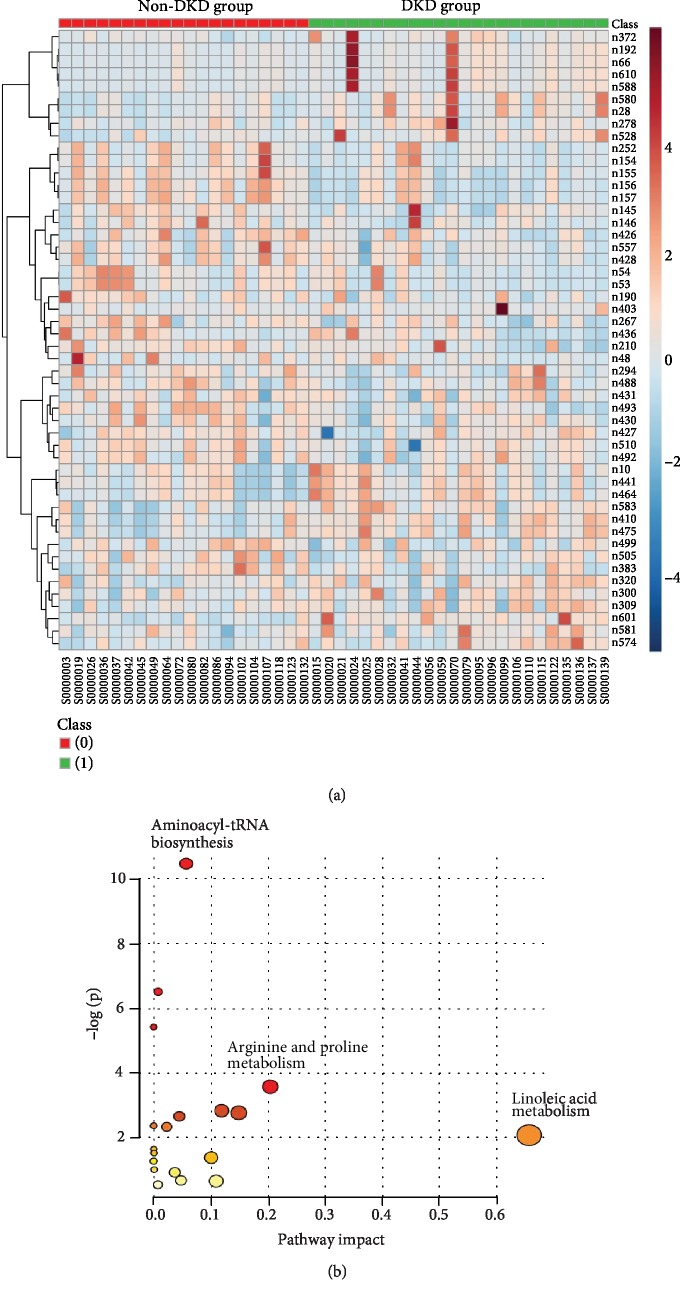
(a) Heatmap showed the differences of metabolics between the DKD group and non-DKD group. (b) The pathway analysis showed that Linoleic Acid metabolism, aminoacyl-tRNA biosynthesis, and arginine and proline metabolism are associated with DKD.

Heatmap and pathway analysis was also performed (Figure 3). The identification of the pathway from the discovery of metabolomics profiling showed that the occurrence and development of DKD would be associated with the linoleic acid metabolism, aminoacyl-tRNA biosynthesis, and arginine and proline metabolism.

### 3.5. Association between Serum Metabolite Intensities and the Presence of DKD

In respect to the associations between DKD and baseline value of the 11 metabolites in the multivariate analysis, all of them exhibited associations with an increased risk of DKD (Table 2). The largest effect size was observed for Piperidine (OR 49.3 per SD increment (5% CI: 4.1, 589.9); *p* < 0.001). It can be seen that the metabolites with protective effects (OR < 1) are on the rise among the DKD group. On the contrary, the risk factors (OR > 1) were declined among the DKD group.

Spearman's correlation analysis between metabolites and UACR showed positive relationships between UACR and Trans-4-Hydroxy-L-Proline, 6-Aminocaproic Acid, L-Dihydroorotic Acid, 6-Methylmercaptopurine, Piperidine, and Cuminaldehyde, whereas negative correlations between UACR and Hexadecanoic Acid (C16:0), Linolelaidic Acid (C18:2N6T), Linoleic Acid (C18:2N6C), Azoxystrobin Acid, and Lysopc 20:4 (Table 3).

**Figure 4:**
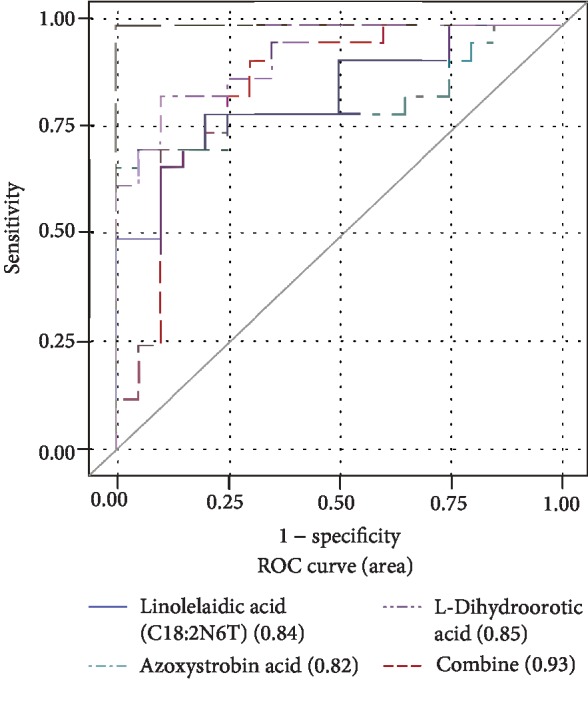
Capability and feasibility of single metabolite and combination (Linolelaidic Acid (C18:2N6T), L-Dihydroorotic Acid, and Azoxystrobin Acid) of three screened metabolites models.

To examine the performance of metabolites in the prediction of DKD, ROC curves were developed. They demonstrated excellent prediction capabilities of prediction in respect to DKD (AUC values > 0.70). Metabolites and associated AUC values are shown in Table 4, while the comparison between different models of ROC analysis is presented in Table 5. L-Dihydroorotic Acid, Linolelaidic Acid (C18:2N6T), Azoxystrobin Acid, and Linoleic Acid (C18:2N6C) exhibited remarkable capabilities of prediction (AUC > 0.80) (Table 4). Furthermore, models were constructed to combine the three metabolites including Linolelaidic Acid (C18:2N6T), L-Dihydroorotic Acid, and Azoxystrobin Acid (AUC values 0.93) as shown in Figure 4.

## 4. Discussion

The major microvascular complications of diabetes include DKD, diabetic encephalopathy, and cardiomyopathy. Being classified as the most lethal diabetic complications, DKD occurs in 20-40% of T2DM. Metabolomics, a novel approach to the identification of the changes in metabolite profiles for biological samples, is extensively used in the treatment of kidney disease [19] and regarded as a valuable tool to study the pathophysiology of chronic kidney disease.

This study is purposed to identify serum metabolic biomarkers to facilitate the detection of DKD from patients' serum samples. In line with the previous studies performed on the association between metabolites with DKD risk, a discovery was made that the extensive metabolic changes were associated with a decline in renal function. In this study, a case-control study was conducted on DKD using a UPLC-ESI-MS/MS-based metabolome analysis to identify the differences in the serum metabolites between non-DKD and DKD samples. The results have demonstrated that OPLS-DA was effective in assisting with this type of analysis. OPLS-DA identified 11 metabolites as major contributors to distinguishing non-DKD from DKD, including lipid-free fatty acid, amino acids, and their derivatives; organic acids and their derivatives; nucleotides and their derivatives; and lipid-fatty acid.

A variety of biological markers have been found to be associated with diabetic kidney disease in recent years, which is significant to predicting the occurrence and development of the disease. Many studies have reported the biomarkers of DKD. There were many studies focusing on the metabolites of diabetic kidney disease, including blood and urine metabolomics studies. Zhang et al. detected significant changes in serum leucine, dihydrosphingosine, and phytic acid sphingosine levels by UPLC coupled with orthogonal acceleration TOFMS (UPLC oa TOF-MS) [20]. Zhu et al. performed metabolic profiling based on normal phase liquid chromatography coupled with TOFMS (NPLC-TOF/MS). Finally, 2 novel biomarkers, PI C18:0/22:6 and SM dC18:0/20:2, were identified to discriminate between healthy individuals, T2DM cases and DKD cases [21]. Van der et al. analyzed the urine profiles of T1DM patients who were clinically defined as having a normal albumin excretion rate using gas chromatography-mass spectrometry (GC-MS) and LC-MS. The discriminating metabolites mainly included acylcarnitines, acylglycines, and metabolites related to tryptophan metabolism [22]. However, few studies have classified the metabolites as measured. Therefore, the measured metabolites were classified by this study.

This study led to a discovery that lipid-free fatty acid metabolite had a close association with DKD (VIP > 6, *p* < 0.001). Hexadecanoic Acid (C16:0), Linolelaidic Acid (C18:2N6T), and Linoleic Acid (C18:2N6C) are invariably classed into lipid-free fatty acid. They all increased among the DKD group and showed protection factor (OR < 1). Moreover, they exhibited excellent capability of prediction for DKD (AUC > 0.80). Linoleic acid is one of the polyunsaturated fatty acids (PUFA) that mediates various metabolic effects, and their effects on metabolism are mainly reliant on inflammation and oxidative stress, which has been well-illustrated in previous reports. Studies have demonstrated that docosahexaenoic acid (DHA) may suppress the expression and secretion of fractalkine through inhibition of the tumor necrosis factor-*α* signaling pathway in DKD patients, which improves inflammation and reduces oxidative stress [23]. Over the years, experiments have indicated that lipids and lipid-derived metabolites were not only involved in inflammatory processes and oxidative stress but also played an important role in the physiological and pathological processes of the kidney. Arachidonic acid (AA) is a component of cell membrane phospholipids, which makes us believe that its connection to the kidney is also related to the improvement of inflammation and reduction of oxidative stress [24]. Studies have found out that low linolenic and linoleic acid consumption are associated with the chronic kidney disease in patients with type 2 diabetes. Moreover, the direct relationship between linoleic acid and diabetic nephropathy remains unclear, which leads to the speculation that it may also play a role in improving inflammation and reducing oxidative stress.

In addition to fat-free fatty acid metabolites, this study revealed that organic acids and their derivatives are also related to DKD. 6-Aminocaproic Acid and L-Dihydroorotic Acid are organic acids; they also bear association with DKD (VIP > 3, *p* ≤ 0.001, AUC > 0.70). Among the 11 metabolites, L-Dihydroorotic Acid exhibited the highest AUC value (0.85, 95% CI: 0.73, 0.98). It has been reported that dihydroorotate dehydrogenase (DHODH) was closely associated with the occurrence and development of various tumors [25]. The inhibition or downregulation of DHODH could help contain tumor cell proliferation [26], induce apoptosis, or enhance the antitumor effect of other target drugs. Diabetes has a close relation to nucleic acid metabolism. DHODH is present in the human mitochondrial inner membrane, which is an iron-containing flavin-dependent enzyme that plays a significant role in the de novo synthesis of pyrimidine. As revealed by previous studies, diabetes was associated with nucleic acid metabolism enzymes [27]. In our view, DHODH has the potential to be a crucial enzyme for L-Dihydroorotic Acid, which is closely associated with the occurrence and development of DKD. A significant increase in Azoxystrobin Acid was observed among DKD patients (*p* < 0.001), indicating the strongest protection in the 11 metabolites. Besides, it also created high AUC values (0.82, 95% CI: 0.69, 0.95). Despite this, it is an unknown species as we know no more than that it is a metabolite of the fungicidal azoxystrobin.

Liu et al. found out that aminoacyl-tRNA biosynthesis, arginine and proline metabolism, phenylalanine, tyrosine, and chromosome metabolomics in plasma of apoA-I knockout mice fed high-fat diet amino acid biosynthesis were affected. This finding suggested that the effects of these metabolic pathways on diabetes are relevant through lipids, which is also consistent with our results [28].

Metabolomics can provide information about how disease processes and what gene function is performed [29]. Plenty of the original works related to diabetes metabolomics were performed using NMR spectroscopy [30, 31]. However, very few studies have been published about the diagnosis and discrimination between serum profiles of DKD and T2DM patients using LC-MS. Compared with human urine commonly used in metabolomics, the blood sample is relatively simpler and showed a smaller variability under normal physiological conditions, which makes it a better choice for the LC-MS metabolite screening. In this work, we employed a metabolomics strategy based on UPLC-ESI-MS/MS to discriminate the serum profiles of DKD patients and T2DM patients. As for the metabolites with low volatility and thermal stability, LC-MS is a powerful alternative that offers high selectivity and sensitivity and has a good potential in diabetes research [32, 33]. This potential has been further enhanced following the introduction of UPLC-MS, with its higher resolution separations [34]. The molecular masses of various compounds can be determined with great accuracy using ESI-MS. Relatively pure samples can be analyzed by direct infusion into ESI-MS after simple dilution and the analytes with interference.

Aside from revealing the potential mechanisms for DKD development, our study also identified 11 metabolic signatures for the prediction of DKD. The combination of Linolelaidic Acid (C18:2N6T), L-Dihydroorotic Acid, and Azoxystrobin Acid contributed to a powerful capability to make a distinction between high-risk group and control. In addition, our results demonstrated that the combination of Linolelaidic Acid (C18:2N6T), L-Dihydroorotic Acid, and Azoxystrobin Acid might serve as a potential indicator of DKD.

### 4.1. Strengths and Limitations

In this study, the 11 metabolites we measured not only have a high statistical significance but also show high predictive values. In addition, the measurement of a wide range of biomarkers in samples was combined to identify the biomarkers with better prediction. It has been demonstrated that there is a possibility to improve the prediction of DKD significantly using three biomarkers in combination. From a technical point of view, this study included an increasing number of studies using MS as a tool to identify biomarkers. However, this study was primarily conducted using LC-ESI-MS/MS to achieve more metabolites. There are also several limitations in this study. Our findings were observed in a single cohort with only small samples of cases and controls. The candidate metabolites from this study ought to be validated in an independent and larger replication sample of Chinese adults. Moreover, the mechanism for the influence of metabolites on DKD needs to be further researched and verified.

## 5. Conclusions

To conclude, an investigation was conducted on the relations between metabolic signatures and the risk of DKD with the application of highly sensitive and complementary MS-based untargeted metabolomics techniques. The present study indicated multiple early metabolic signs for DKD, which were conducive to advancing prediction and prevention of DKD among the Chinese populations. A total of 11 new metabolites bearing close association with DKD were identified. The combination of Linolelaidic Acid (C18:2N6T), L-Dihydroorotic Acid, and Azoxystrobin Acid especially represented a potential indicator of diabetes progress. Further studies, especially large-scale independent validation, may be necessary.

## Figures and Tables

**Table 1:** Demographic and clinical characteristics of participants.

Variables	DKD	Non-DKD	*p* value
*N*	24	20	
Age (years)	58.00 ± 8.99	57.05 ± 9.16	0.731
Male, # (%)	12 (50)	10 (50)	1
HbA1C (%)	9.71 ± 1.78	9.28 ± 1.72	0.419
BMI (kg/m^2^)	24.33 ± 2.87	24.99 ± 3.73	0.51
Duration (years)	11.75 ± 5.62	8.92 ± 6.24	0.122
Systolic blood pressure (mmHg)	133.33 ± 15.43	126.35 ± 13.00	0.116
Diastolic blood pressure (mmHg)	77 (73, 80)	78 (74, 84)	0.369
Fasting plasma glucose (mmol/L)	8.22 ± 3.21	8.49 ± 3.00	0.776
Postprandial glucose (mmol/L)	16.80 ± 3.50	16.71 ± 4.52	0.947
C-peptide (ng/mL)			
0 minute	1.38 ± 1.35	1.45 ± 1.76	0.895
120 minutes	3.21 ± 2.42	4.49 ± 3.38	0.207
Low-density lipoprotein cholesterol (mmol/L)	2.24 ± 0.88	2.62 ± 1.07	0.2
High-density lipoprotein cholesterol (mmol/L)	0.91 ± 0.23	1.15 ± 0.42	0.02
Triglyceride (mmol/L)	2.05 ± 2.11	2.06 ± 1.20	0.977
Total cholesterol (mmol/L)	4.37 ± 1.29	5.02 ± 1.03	0.075
Creatinine (*μ*mol/L)	61.7 (51.9, 77.3)	58.8 (47.3, 70.0)	0.39
UACR (mg/mmol)	22.10 (6.47, 138.40)	2.04 (1.48, 2.61)	<0.001
Estimated glomerular filtration rate	93.74 ± 24.18	100.16 ± 10.0	0.245
Uric acid (*μ*mol/L)	334.26 ± 74.24	328.40 ± 89.24	0.815
24-hour urinary protein (g/24 h)	0.18 (0.78, 0.87)	0.04 (0.02, 0.068)	<0.001
Free triiodothyronine (pg/mL)	3.19 ± 0.39	2.97 ± 0.31	0.061
Free thyroxine (ng/dL)	1.22 ± 0.15	1.22 ± 0.17	0.967
Thyroid stimulating hormone (*μ*IU/mL)	2.0 (1.41, 2.29)	1.78 (1.0, 2.18)	0.443
*β*-CTX (pg/mL)	415.91 ± 201.04	334.55 ± 213.43	0.231
N-MID (ng/mL)	12.79 ± 5.47	11.09 ± 4.25	0.285
T-PINP (ng/mL)	39.6 ± 16.76	35.75 ± 14.9	0.453
Vitamin D (ng/mL)	17.78 ± 8.55	21.23 ± 6.75	0.184
Fatty liver, # (%)	14 (58.3)	14 (70)	0.07
Smoking, # (%)	5 (21)	4 (20)	0.946
Drinking, # (%)	7 (29)	3 (15)	0.264

Continuous data met normal or similar normal distribution were presented as mean ± standard deviation (SD) and compared with independent *t*-test. Otherwise, they were described as median (1st percentile, 3rd percentile) and Mann–Whitney *U* tests were performed for the comparisons between the cases and controls. Categorical data were presented as frequency (percentage), and chi-square tests were applied to compare the differences between the two groups.

**Table 2:** The statistical difference in 11 serum metabolic biomarker candidates.

Metabolites	Class	VIP	*p* value	Trend	Mode	Rt	*M*/*Z*	OR (95% CI)	*p* value	FDR-adjusted *p* value
Hexadecanoic Acid (C16:0)	Lipid-free fatty acid	6.02239	<0.001	UP	N	11.65	256.24	0.2 (0.1, 0.5)	0.002	0.028
Linolelaidic Acid (C18:2N6T)	Lipid-free fatty acid	6.25109	<0.001	UP	N	11.1	280.24	0.2 (0.1, 0.5)	<0.001	0.016
Linoleic Acid (C18:2N6C)	Lipid-free fatty acid	6.05045	<0.001	UP	N	11.1	280.24	0.2 (0.1, 0.6)	0.001	0.018
Trans-4-Hydroxy-L-Proline	Amino acids and their derivatives	4.10297	0.004	Down	P	1.21	131.058	2.4 (1.3, 4.5)	0.006	0.058
6-Aminocaproic Acid	Organic acids and their derivatives	3.67465	0.001	Down	P	1.16	131.095	4.3 (1.8, 10.5)	0.001	0.028
L-Dihydroorotic Acid	Organic acids and their derivatives	4.06423	<0.001	Down	P	0.58	158.033	35.0 (4.0, 308.4)	0.001	0.018
6-Methylmercaptopurine	Nucleotides and their derivatives	3.08662	0.005	Down	P	1.85	166.0313	2.7 (1.3, 5.8)	0.009	0.065
Piperidine	—	1.53861	0.002	Down	P	1.16	85.08915	49.3 (4.1, 589.9)	0.002	0.04
Azoxystrobin Acid	—	1.18869	<0.001	UP	P	6.99	389.1012	0.0 (0.0, 0.0)	0.002	0.001
Lysopc 20:4	Lipid-fatty acid	6.07644	0.002	UP	P	9		0.4 (0.2, 0.7)	0.004	0.03
Cuminaldehyde	Lipid-fatty acid	1.3433	0.002	Down	P	1.87	148.0888	14.9 (2.2, 101.8)	0.006	0.04

VIP: variable importance in the project; N: negative mode; P: positive mode; Rt: retention time (minutes); *M*/*Z*: mass-charge ratio; FDR: false discovery rate-adjusted *p* value.

**Table 3:** Correlation analysis between UACR and metabolites.

Metabolite	UACR	
	*r*	*p* value
Hexadecanoic Acid (C16:0)	-0.521	<0.001
Linolelaidic Acid (C18:2N6T)	-0.55	<0.001
Linoleic Acid (C18:2N6C)	-0.525	<0.001
Trans-4-Hydroxy-L-Proline	0.356	0.018
6-Aminocaproic Acid	0.446	0.002
L-Dihydroorotic Acid	0.597	<0.001
6-Methylmercaptopurine	0.356	0.018
Piperidine	0.399	0.007
Azoxystrobin Acid	-0.564	<0.001
Lysopc 20:4	-0.363	0.015
Cuminaldehyde	0.385	0.01

UACR: urinary albumin/creatinine ratio.

**Table 4:** Capability and feasibility of each metabolite in distinguishing DKD from non-DKD based on ROC analysis.

Models	AUC	95% CI	SE
Hexadecanoic Acid (C16:0)	0.8	0.66, 0.93	0.07
Linolelaidic Acid (C18:2N6T)	0.84	0.72, 0.95	0.06
Linoleic Acid (C18:2N6C)	0.81	0.68, 0.94	0.07
Trans-4-Hydroxy-L-Proline	0.74	0.58, 0.89	0.08
6-Aminocaproic Acid	0.79	0.65, 0.93	0.07
L-Dihydroorotic Acid	0.85	0.73, 0.98	0.06
6-Methylmercaptopurine	0.73	0.58, 0.88	0.08
Piperidine	0.75	0.60, 0.90	0.08
Azoxystrobin Acid	0.82	0.69, 0.95	0.07
Lysopc 20:4	0.75	0.60, 0.90	0.08
Cuminaldehyde	0.76	0.61, 0.90	0.07
Combine	0.93	0.85, 1.00	0.04

AUC: area under the curve.

**Table 5:** Comparison between different models of ROC analysis.

Models	*p* values
Linolelaidic Acid (C18:2N6T)_Combine	0.0065
L-Dihydroorotic Acid_Combine	0.00194
Azoxystrobin Acid_Combine	0.0076
L-Dihydroorotic Acid_Linolelaidic Acid (C18:2N6T)	0.8192
Azoxystrobin Acid_Linolelaidic Acid (C18:2N6T)	0.8194
Azoxystrobin Acid_L-Dihydroorotic Acid	0.6862
Linolelaidic Acid (C18:2N6T)_combine1	0.0886
L-Dihydroorotic Acid_combine1	0.13
Azoxystrobin Acid_combine1	0.0502

The model of Combine 1 contains Linolelaidic Acid (C18:2N6T), L-Dihydroorotic Acid, and Azoxystrobin Acid; the model of Combine contains 11 metabolites. Chi-square test was utilized to compare the differences.

## Data Availability

The data used to support the findings of this study are available from the corresponding author upon request.
